# Combined Use of Electrocardiography and Ultrasound to Detect Cardiac and Pulmonary Involvement after Recovery from COVID-19 Pneumonia: A Case Series

**DOI:** 10.3390/jcdd8100133

**Published:** 2021-10-17

**Authors:** Jacopo Marazzato, Roberto De Ponti, Paolo Verdecchia, Sergio Masnaghetti, Dina Visca, Antonio Spanevello, Monica Trapasso, Martina Zappa, Antonella Mancinelli, Fabio Angeli

**Affiliations:** 1 Department of Medicine and Surgery, University of Insubria, 21100 Varese, Italy; j.marazzato88@gmail.com (J.M.); roberto.deponti@uninsubria.it (R.D.P.); dina.visca@uninsubria.it (D.V.); antonio.spanevello@uninsubria.it (A.S.); marty-italy92@hotmail.it (M.Z.); antonella.mancinelli@yahoo.it (A.M.); 2 Fondazione Umbra Cuore e Ipertensione-ONLUS and Division of Cardiology, Hospital S. Maria della Misericordia, 06100 Perugia, Italy; verdecchiapaolo@gmail.com; 3 Department of Medicine and Cardiopulmonary Rehabilitation, Maugeri Care and Research Institute, IRCCS Tradate, 21049 Tradate, Italy; sergio.masnaghetti@icsmaugeri.it; 4 Dipartimento di Igiene e Prevenzione Sanitaria, PSAL, Sede Territoriale di Varese, ATS Insubria, 21100 Varese, Italy; montrapasso@gmail.com

**Keywords:** SARS-CoV-2, COVID-19, interstitial pneumonia, long COVID, electrocardiography, transthoracic echocardiography, lung ultrasound, diaphragmatic thickness, diaphragmatic excursion

## Abstract

Background: Although severe acute respiratory syndrome coronavirus-2 (SARS-CoV-2) may cause an acute multiorgan syndrome (coronavirus disease 2019 (COVID-19)), data are emerging on mid- and long-term sequelae of COVID-19 pneumonia. Since no study has hitherto investigated the role of both cardiac and pulmonary ultrasound techniques in detecting such sequelae, this study aimed at evaluating these simple diagnostic tools to appraise the cardiopulmonary involvement after COVID-19 pneumonia. Methods: Twenty-nine patients fully recovered from COVID-19 pneumonia were considered at our centre. On admission, all patients underwent 12-lead electrocardiogram (ECG) and transthoracic echocardiography (TTE) evaluation. Compression ultrasound (CUS) and lung ultrasound (LUS) were also performed. Finally, in each patient, pathological findings detected on LUS were correlated with the pulmonary involvement occurring after COVID-19 pneumonia, as assessed on thoracic computed tomography (CT). Results: Out of 29 patients (mean age 70 ± 10 years; males 69%), prior cardiovascular and pulmonary comorbidities were recorded in 22 (76%). Twenty-seven patients (93%) were in sinus rhythm and two (7%) in atrial fibrillation. Persistence of ECG abnormalities from the acute phase was common, and nonspecific repolarisation abnormalities (93%) reflected the high prevalence of pericardial involvement on TTE (86%). Likewise, pleural abnormalities were frequently observed (66%). TTE signs of left and right ventricular dysfunction were reported in two patients, and values of systolic pulmonary artery pressure were abnormal in 16 (55%, despite the absence of prior comorbidities in 44% of them). Regarding LUS evaluation, most patients displayed abnormal values of diaphragmatic thickness and excursion (93%), which correlated well with the high prevalence (76%) of pathological findings on CT scan. CUS ruled out deep vein thrombosis in all patients. Conclusions: Data on cardiopulmonary involvement after COVID-19 pneumonia are scarce. In our study, simple diagnostic tools (TTE and LUS) proved clinically useful for the detection of cardiopulmonary complications after COVID-19 pneumonia.

## 1. Introduction

Since the initial outbreak in Wuhan, China, in December 2019, severe acute respiratory syndrome coronavirus-2 (SARS-CoV-2) infection and its related syndrome (coronavirus disease 2019 (COVID-19)) have spread further afield, leading to a globalised pandemic and a significant disruption of our daily lives [1].

Although the virus is well known to lead to a variety of multiorgan clinical manifestations during the acute phase [1,2,3,4,5], preliminary clinical [6] and radiological [7] data have recently reported mid-term cardiopulmonary sequelae in fully recovered patients.

However, no study has hitherto focused attention on the combined role of different ultrasound techniques in assessing cardiac or pulmonary involvement after recovery from COVID-19 pneumonia.

Therefore, the aim of this study was to evaluate echocardiographic (transthoracic echocardiography (TTE)) features, possibly related to electrocardiographic (ECG) abnormalities, in patients with previous COVID-19 pneumonia and to elucidate possible cardiovascular involvement; furthermore, using compression ultrasound (CUS) and lung ultrasound (LUS), we explored the presence of deep venous thrombosis (DVT) and/or lung abnormalities related to COVID-19 pneumonia.

## 2. Materials and Methods

In the setting of an ongoing prospective registry, patients affected by COVID-19 were consecutively considered at our Department of Medicine and Cardiopulmonary Rehabilitation of the Maugeri Care and Research Institute of Tradate-Varese, Italy [8,9,10]. Inclusion criteria included full recovery from COVID-19 pneumonia and immediate referral to our study centre to undergo a full cardiopulmonary rehabilitation and therapy optimisation program. Before admission, at least two negative nasopharyngeal swabs were required for each patient, together with clinical, laboratory and instrumental stability. The majority of patients were transferred from other hospitals of our network. The hospital staff of our department included internists, cardiologists, pneumologists and physiotherapists. This study conformed to the Declaration of Helsinki on human research and was approved by the ethical committee at our institution. Patients or their legal guardians signed informed consent.

Upon admission, a thorough clinical evaluation assessing each patient’s prior comorbidities was carried out, along with laboratory and arterial blood gas tests (at admission and at discharge). For the latter, a PaO_2_/FiO_2_ ratio of <200 was deemed abnormal [11]. Moreover, 12-lead ECG, TTE and LUS were all performed within the first week of hospitalisation to appraise potential cardiac and pulmonary involvement. DVT was ruled out by means of CUS.

ECG analysis and data collection have been described elsewhere [10,11]. In brief, ECG was recorded with 25 mm/s and 1 mV/cm calibration and a 0.05–150 Hz filter setting. ECG tracings were coded and were analysed off-line. The following ECG data were measured: heart rate (HR), correct QT interval (ms) and the presence of ST-T abnormalities according to both the Minnesota coding [11,12] (yes vs. no) and qualitative evaluation. The QT interval was measured from Q wave outset to T wave termination and corrected by the HR using Bazett’s formula. Measures of the PR interval and the QRS complex were also included in the final analysis, together with the presence and type of arrhythmia, when identified. Main ECG changes related to cardiovascular complications were classified according to current guidelines [13,14].

TTE was performed by the same operator using the same device (GE Vivid E9 ultrasound machine). Images were acquired in two-dimensional (2D) echocardiography using bi-dimensional (B)-mode, mono-dimensional (M)-mode, continuous-wave (CW) Doppler, pulsate-wave (PW) Doppler and colour Doppler [15]. In addition to the full assessment of left ventricular function and segmental wall motion abnormalities, as appraised by the left ventricular ejection fraction (LVEF, %; apical four-chamber view, apical two-chamber view, biplane method, Simpson’s rule) and the wall motion score index (WMSI; long- and short-axis view) [15], valve diseases were also evaluated and graded from mild to severe using apical four-chamber view and colour Doppler [16]. Moreover, significant attention was devoted to the evaluation of the right-sided chambers of the heart and the ensued alterations potentially occurring after COVID-19 pneumonia. In particular, the following TTE parameters were considered: right atrial area (RAA; normal values ≤ 18 cm^2^; apical four-chamber view, B-mode) [17], right ventricular end-diastolic basal diameter (RVEDD; normal values ≤ 41 mm; apical four-chamber view, B-mode) [15], right ventricular longitudinal systolic function using the tricuspid annular plane systolic excursion (TAPSE; normal values > 17 mm; apical four-chamber view, M-mode; apical four-chamber view, B-mode) [15], inferior vena cava (IVC) size (normal values < 21 mm) as an estimate of the right atrial pressure (RAP; subcostal view, B-mode, M-mode) [15] and systolic pulmonary artery pressure (SPAP, mmHg; apical four-chamber view, CW Doppler) measured using the simplified Bernoulli formula. Abnormal values were considered for PAPS > 30 mmHg, and pulmonary hypertension was suspected at tricuspid regurgitation velocity (TRV) > 2.8–2.9 ms and SPAP > 36 mmHg [18]. Data regarding plural and pericardial features (presence, yes vs. no, maximum diameter, presence of thickening and hyper-echogenicity, yes vs. no; apical four-chamber view, long- and short-axis view, subcostal view; B-mode) were also collected [15].

To conclude our evaluation, we performed CUS and LUS to rule out DVT and pulmonary involvement. The methodology for LUS and the meaning of the investigated parameters have been reported elsewhere [19,20]. Briefly, using conventional-brightness B-mode and M-mode ultrasound with a low-frequency (2–5 MHz) curvilinear probe, left- and right-sided sliding (yes vs. no) [20], B-lines (yes vs. no) [20] and left- and right-sided curtain sign (yes vs. no) [19] were all evaluated. Finally, the bilateral hemi-diaphragmatic thickness (DT) ratio and diaphragmatic excursion (DE) were assessed [21,22]. The DT ratio, expressed as DT at rest divided by DT at total lung capacity, was abnormal if <2 [22] regardless of sex. DE (mm) was evaluated at maximal inspiration for each patient and considered within normal values if >47 mm and >37 mm for men and women, respectively [21]. Finally, radiological data of chronic lung damage detected on the thoracic computed tomography (CT) scan (such as bronchiectasis, emphysema and pulmonary fibrosis) were collected and correlated with specific LUS parameters.

We used STATA 16 (StataCorp, College Station, TX, USA) and R software version 3 for data analysis. In case series analysis, continuous variables were expressed as the mean (±standard deviation); categorical variables were expressed as a number (%). We used the paired sample t-test to analyse pairs of observations and to determine whether the mean difference between two sets of observations is zero.

## 3. Results

### 3.1. Patient Population

After full recovery from COVID-19 pneumonia, 29 patients (mean age 70 ± 10 years; males 69%) were consecutively enrolled. The patients’ known comorbidities and COVID-19 clinical management are reported in Table 1. Twenty-one patients had a history of hypertension (72%), while 20 (69%), 8 (28%) and 9 (31%) suffered from diabetes mellitus (DM), obesity and chronic obstructive pulmonary disease (COPD), respectively (Table 1). Prior to SARS-CoV-2 infection, 13 patients (45%) were known for cardiac and cerebrovascular comorbidities: coronary artery disease (CAD) and atrial fibrillation (AF) in 10 (34%), stroke in 2 (7%) and prior hospitalisations for heart failure (HF) in 1 patient (3%).

**Figure 1:**
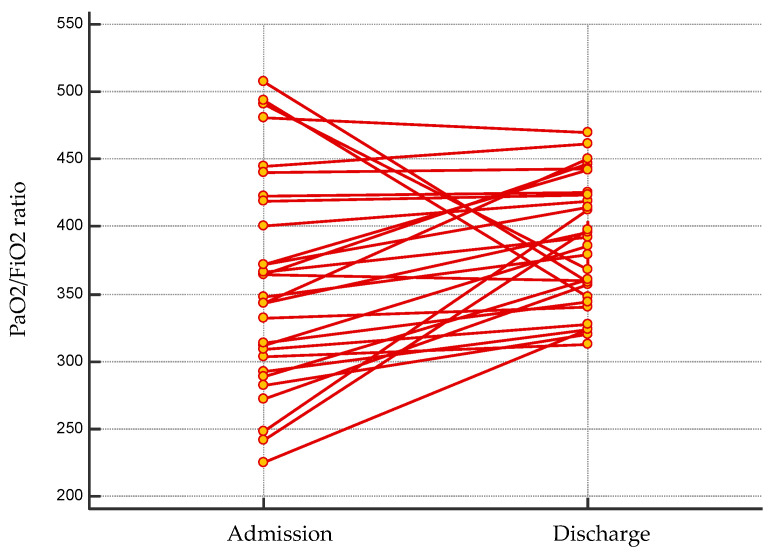
Changes in PaO_2_/FiO_2_ ratio from admission to discharge.

Before being referred to our study centre, all patients were managed for COVID-19 pneumonia apart from one case (3%) treated for virus-related pulmonary embolism. In-hospital stay was 37 ± 14 days on average. During the acute phase of COVID-19, in addition to oxygen therapy (29; 100%), non-invasive ventilation (NIV) and/or endotracheal intubation (ETI) was deemed necessary in 10 cases (35%). Medical therapy was required in 26 (90%), as follows: antiretroviral therapy, hydroxychloroquine, steroids and tocilizumab in 18 (62%), 24 (83%), 11 (38%) and 3 (10%) patients, respectively (Table 2). On admission, the arterial blood gas test was performed for each patient and the mean PaO_2_/FiO_2_ ratio was 358 ± 78. The average length of stay at our rehabilitation centre was 21 ± 6 days. At discharge, a significant average increase in PaO_2_/FiO_2_ ratio was documented (358 ± 78 vs. 386 ± 47, *p* = 0.048; Figure 1). Cardiac and pulmonary complications recorded during hospitalisation were treated according to current guidelines.

### 3.2. Electrocardiographic Findings

On admission (Table 3), most patients (27; 93%) were in sinus rhythm (SR; mean heart rate (HR): 80 ± 15 bpm); AF was documented in 2 patients (7%): this arrhythmia developed during the acute phase of infection (HR: 80 ± 15 bpm on average).

In patients in SR, the average PR interval was 167 ± 35 ms and first-degree AV block was recorded in three patients (10%). The mean QRS complex duration was 98 ± 12 ms, with evidence of left anterior fascicular block in four hypertensive patients (14%); low voltage and mild ST-T abnormalities were commonly observed (41% and 93%, respectively), even in cases without prior structural heart disease.

Furthermore, the QTc interval was significantly prolonged in three patients (10%) treated with amiodarone.

Remarkably, the majority of abnormal ECG features (Table 3) developed during the acute phase of infection and were classified as persistent ECG features of COVID-19 pneumonia.

### 3.3. Transthoracic Echocardiogram

The mean LVEF was 65% ± 7%. No significant wall motion abnormalities were observed in most patients, with the only exception of one case (3%) with a prior history of CAD and showing mildly reduced LVEF (47%) along with an abnormal WMSI (73). Interestingly, no signs of structural heart disease had been previously recorded in this patient.

Significant mitral and/or tricuspid regurgitation was reported in less than one-third of the investigated population (8; 28%).

As to right-sided parameters, these are reported in Table 3. Although the right atrium (RA) was enlarged in 6 (21%) patients, right ventricular (RV) size (mean RVEDD 32 ± 3 mm) and longitudinal systolic function (TAPSE 23 ± 3 mm) fell within normal values in most cases (28; 97%). However, despite no clear signs of RA or RV enlargement, a mildly reduced TAPSE was observed in only one patient (Table 3) without any prior comorbidity (Table 1).

Of note, high SPAP values were recorded in about half of the evaluated patients (16; 55%) and were significantly increased in 9 (31%), even without any prior cardiac or pulmonary disease in up to 44% of them.

**Figure 2:**
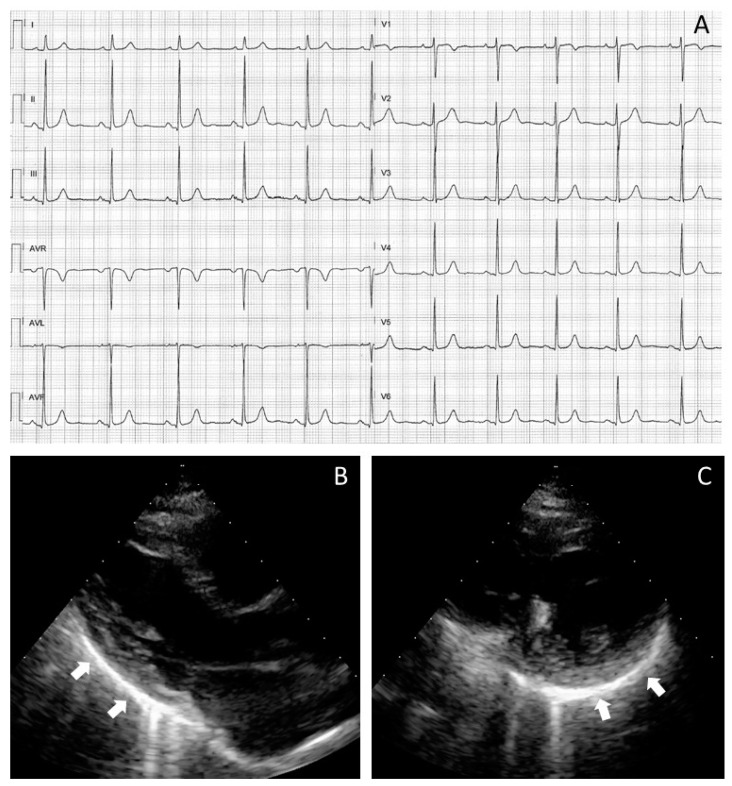
Cardiac manifestation in a patient fully recovered from COVID-19 pneumonia and with pericardial effusion developed during the acute phase of infection. The 12-lead electrocardiogram shows nonspecific repolarisation abnormalities on the inferior and right precordial leads (**A**). Different echocardiographic projections (parasternal long axis view in (**B**) and parasternal short axis view in (**C**)) display sequelae of a previous acute pericarditis (pericardial thickening and a dazzling hyper echogenicity with comet-like artefacts). Such TTE signs are more evident on the infero-lateral wall of the left atrium (arrows) with no sign of pericardial effusion.

Finally, pericardial abnormalities were commonly observed after SARS-CoV2 pneumonia as pericardial thickening and/or hyper-echogenicity (19 patients; 66%), low-to-mild pericardial effusion (20 patients; 69%; 3 ± 3 mm on average), or both (24 patients; 83%). As exemplified in Figure 2, the pericardial involvement on TTE reflected the high prevalence of mild ST-T changes on ECG in most patients (23; 79%).

### 3.4. Compression Ultrasonography, Lung Ultrasound and Thoracic CT Scan Findings

For each patient, no signs of DVT were identified at CUS. Parameters evaluated at LUS are reported in Table 4.

As observed for pericardial involvement on TTE, pleural abnormalities were also common in the investigated population (66%): pleural effusion, thickening and/or hyper-echogenicity or both were recorded in 41%, 59% and 66% of patients, respectively. However, pleural sliding was reduced in two patients only (7%) (Table 4).

No signs of alveolar thickening (B-lines) were observed, and a bilateral good expansion of the basal portion of the lungs (curtain sign) was generally reported (Table 4).

However, despite being fully recovered from COVID-19 pneumonia, the vast majority of the enrolled patients displayed an abnormal DT ratio and DE (27; 93%), which correlated well with the evidence of sub-acute/chronic radiological findings of lung damage, such as bronchiectasis (17; 63%), emphysema (15; 56%) and/or pulmonary fibrosis (11; 41%), as displayed in Table 4.

## 4. Discussion

Although acute clinical presentation is well known, long-term COVID-19-related symptoms have been recently described [23,24] and have progressively drawn the attention of the scientific community worldwide.

Ultrasound imaging of the heart and lung and associated tissues may play an important role in the management of patients with COVID-19-associated organ injury [25]. However, data on long-term cardiopulmonary involvement occurring after COVID-19 are scarce, and to our knowledge, no study has thoroughly investigated the combined role of 12-lead ECG, TTE, CUS and LUS in this clinical scenario so far.

In a case series of 29 patients hospitalised for cardiopulmonary rehabilitation and therapy optimisation after recovery from SARS-CoV-2-related pneumonia, we showed that, regardless of prior comorbidities, cardiac and pulmonary involvement occurring after COVID-19 is common and may be easily identified by the combined use of promptly available and easy-to-use diagnostic tools.

As reported by a large body of evidence, besides interstitial pneumonia, SARS-CoV-2 may be responsible for a cardiac phenotype of the syndrome, potentially leading to new-onset atrial fibrillation, pulmonary embolism and non-ST elevation myocardial infarction [2,3,5,10,11,26]. However, the virus may elicit a far less dramatic clinical presentation with unremarkable electrocardiographic abnormalities, such as mild ST-T changes [10,27]. Of note, most of these new-onset ECG findings are recorded in patients with negative nasopharyngeal swabs and several weeks after recovery from COVID-19 pneumonia [10]. In keeping with these observations, ECG abnormalities were commonly reported in the population investigated in this study as persistent manifestations of previous COVID-19 pneumonia and reflected the high prevalence of the pericardial involvement observed on TTE.

It is indeed well known that SARS-CoV-2 infection may elicit a powerful multiorgan inflammatory response [2,3,4,28,29,30,31] causing pleural and pericardial inflammation in the affected cases [32]. If the post-mortem analysis of these patients [33] showed that mild pericardial effusion was extremely common immediately after the acute phase of the disease, mounting evidence has recently reported how pericardial involvement may be identified on cardiac magnetic resonance (CMR), even in fully recovered cases [24]. Likewise, ongoing myocardial inflammation and residual fibrosis may still be detectable on CMR as areas of myocardial oedema and/or late gadolinium enhancement [24]. However, as demonstrated in this study, LUS and TTE can be useful in the detection of pleural and pericardial abnormalities, such as pleuro-pericardial thickening, hyper echogenicity and effusion.

Furthermore, LV and RV size and function may also be easily appraised. Among the investigated cases, mildly reduced LVEF was recorded in only one, probably on account of the synergistic effect that CAD and SARS-CoV-2 might have had on LV dysfunction in this patient [11]. However, SPAP values were significantly increased in roughly half of the appraised cases with no association with prior cardiac or pulmonary diseases, RV enlargement or reduced TAPSE, except for one patient for the latter.

Increased RV afterload occurring after SARS-CoV-2-related venous thromboembolism (VTE) [34] or parenchymal lung damage [35] may be deemed responsible for these findings.

Apart from one patient, none of the investigated cases reported signs of pulmonary embolism on thoracic CT scan nor DVT on CUS. Conversely, at least 70% of them displayed radiological sequelae of chronic lung injury. Indeed, a variety of CT lung abnormalities have been reported in patients after COVID-19 pneumonia [7], from ground glass areas to signs of reticulation and parenchymal distortion occurring up to 6 months after acute infection [7]. Although prolonged mechanical ventilation and direct or indirect viral-mediated injury might be associated with post-COVID-19 lung disease [7], in our study 73% of patients not requiring NIV and/or ETI during the acute phase showed signs of emphysema, bronchiectasis and/or fibrosis on CT scan as soon as a few weeks from full recovery. Moreover, these radiological abnormalities were clearly associated with an altered DT ratio and/or DE on LUS, and more importantly, signs of diaphragmatic thickness/excursion were clearly abnormal, even in patients with no radiological evidence of pulmonary sequelae.

Although LUS diaphragmatic excursion proved to predict successful weaning in critically ill patients during the acute phase of the disease [36], to the best of our knowledge, this is the first study to evaluate DT and DE values in fully recovered patients. Considering the proven role of these echo parameters in assessing a decreased exercise capacity and increased dyspnoea in COPD patients [37], they might as well predict the outcome of patients after COVID-19 pneumonia. However, considering the contrasting evidence on the results of pulmonary function tests after SARS-CoV-2-related pneumonia [38,39], prospective and more powered studies are mandatory to define the abnormal thresholds, feasibility and diagnostic power of such echo imaging techniques in this patient population. Finally, future research is needed to determine the long-term persistence of COVID-19-related cardiac and pulmonary sequelae, their clinical significance and impact on these patients and the potential modalities to prevent and treat such potentially life-threatening consequences [40].

## 5. Conclusions

Although acute pulmonary and extra-pulmonary manifestations of SARS-CoV-2 infection are well known, data on long-term cardiac and pulmonary involvement after COVID-19 pneumonia are scarce. Despite the great diagnostic accuracy of CMR and thoracic CT scan to detect both heart and lung involvement, in our study TTE and LUS (which are representative non-invasive methods of examination) proved useful to identify cardiac and pulmonary involvement in patients fully recovered from COVID-19 pneumonia.

In our experience, echo evidence of pleural and pericardial involvement, together with signs of reduced diaphragmatic thickness and excursion, is significantly common in recovered patients and well correlated with parenchymal lung damage on thoracic CT scan. Adequately powered studies are required to clarify the clinical significance and/or impact of these long-term sequelae.

### Study Limitations

Being an explorative analysis, this study should be interpreted within the context of its potential limitations.

First, because 100% of our patients were whites, results may not be extrapolated to different ethnic groups.

Second, during data collection, concern was raised that pulmonary function testing could represent a potential avenue for COVID-19 transmission. Thus, pulmonary function tests were not routinely performed. Moreover, TTE, CUS and LUS were not performed routinely during the acute phase of COVID-19, and we were unable to clearly evaluate serial changes in cardiac and pulmonary injuries.

Finally, our data estimate the severity of COVID-19 only by the need for ventilatory support or oxygen delivery and our protocol did not include a follow-up evaluation after discharge.

## Figures and Tables

**Table 1:** Comorbidities (pulmonary and cardiovascular comorbidities before SARS-CoV-2 infection) in the investigated population.

			Comorbidities							
Pt.	Sex	Age (Years)	Obesity ^a^	COPD	CAD	CVD	HF	AF	DVT	Acute Pericarditis
1	M	57	-	-	-	-	-	-	-	-
2	M	74	-	-	-	-	-	-	-	-
3	M	83	Yes	Yes	-	-	-	-	-	-
4	F	78	Yes	-	-	-	-	-	-	-
5	M	71	-	-	-	-	-	-	-	Yes
6	F	85	-	-	-	-	-	Yes	-	-
7	M	58	Yes	-	-	-	-	-	-	-
8	M	82	-	-	-	Yes	-	Yes	-	-
9	F	74	-	-	-	-	-	-	Yes	-
10	F	79	Yes	-	Yes	Yes	Yes	-	-	-
11	M	73	-	-	-	-	-	-	-	-
12	M	62	-	Yes	-	-	-	-	-	-
13	M	59	-	-	-	-	-	-	-	-
14	F	84	-	-	-	-	-	-	-	-
15	M	58	Yes	-	-	-	-	-	-	-
16	M	59	-	-	Yes	-	-	-	-	-
17	F	78	-	-	-	-	-	-	-	-
18	M	71	-	-	Yes	-	-	-	-	-
19	M	54	-	-	-	-	-	-	-	-
20	M	65	-	-	-	-	-	-	-	-
21	M	75	-	-	-	-	-	Yes	-	-
22	M	75	Yes	Yes	-	-	-	Yes	-	-
23	M	68	-	-	-	-	-	-	-	-
24	F	79	-	-	-	-	-	-	-	-
25	M	52	Yes	-	-	-	-	-	-	-
26	F	50	Yes	-	-	-	-	-	-	-
27	F	81	-	-	-	-	-	Yes	-	-
28	M	70	Yes	-	Yes	-	-	-	-	-
29	M	77	-	-	Yes	-	-	-	-	-

**^a^** Obesity defined for BMI > 30 kg/m^2^. AF, atrial fibrillation; ARV, antiretroviral drugs; CAD, coronary artery disease; COPD, chronic obstructive pulmonary disease; CVD, cerebrovascular disease; DVT, deep vein thrombosis; HF, heart failure; Pt, patient.

**Table 2:** Management of SARS-CoV-2-related pneumonia in the investigated population.

			COVID-19-Related Clinical Presentation and Management						
Pt.	Sex	Age (Years)	Pneumonia	Pulmonary Embolism	NIV/ETI	ARV	HCQ	Steroids	Tocilizumab
1	M	57	Yes	-	-	Yes	Yes	Yes	-
2	M	74	Yes	-	-	-	Yes	-	-
3	M	83	Yes	-	Yes	Yes	Yes	Yes	-
4	F	78	Yes	-	-	-	Yes	-	-
5	M	71	Yes	-	Yes	Yes	Yes	Yes	Yes
6	F	85	Yes	-	-	-	-	-	-
7	M	58	Yes	-	Yes	Yes	Yes	Yes	-
8	M	82	Yes	-	-	Yes	Yes	-	-
9	F	74	Yes	-	-	Yes	Yes	-	-
10	F	79	Yes	Yes	-	Yes	Yes	-	-
11	M	73	Yes	-	-	Yes	Yes	-	-
12	M	62	Yes	-	Yes	-	Yes	-	-
13	M	59	Yes	-	Yes	Yes	Yes	-	-
14	F	84	Yes	-	-	Yes	Yes	-	-
15	M	58	Yes	-	Yes	Yes	Yes	-	-
16	M	59	Yes	-	Yes	Yes	Yes	-	-
17	F	78	Yes	-	-	-	Yes	-	-
18	M	71	Yes	-	Yes	-	Yes	Yes	-
19	M	54	Yes	-	-	-	-	Yes	Yes
20	M	65	Yes	-	Yes	-	-	-	-
21	M	75	Yes	-	-	-	-	-	-
22	M	75	Yes	-	Yes	Yes	Yes	Yes	-
23	M	68	Yes	-	-	Yes	Yes	-	-
24	F	79	Yes	-	-	-	Yes	Yes	-
25	M	52	Yes	-	Yes	Yes	Yes	Yes	Yes
26	F	50	Yes	-	Yes	Yes	Yes	-	-
27	F	81	Yes	-	Yes	-	Yes	-	-
28	M	70	Yes	-	-	Yes	Yes	Yes	-
29	M	77	Yes	-	Yes	Yes	-	Yes	-

ARV, antiretroviral drugs (different combinations of antiretroviral drugs were used, as follows: darunavir/ritonavir, darunavir/cobicistat and lopinavir/ritonavir); ETI, endotracheal intubation; HCQ, hydroxychloroquine; NIV, non-invasive ventilation; Pt, patient.

**Table 3:** Electrocardiographic and echocardiographic features of patients fully recovered from COVID-19 pneumonia.

	Abnormal ECG Features		Transthoracic Echocardiogram							
Pt.	Recognised at Admission	Changes from the Acute Phase	RAA (cm^2^)	RVEDD (mm)	TAPSE (mm)	TR (grade)	IVC (mm)	SPAP (mmHg)	Pericardial Effusion	Pericardial Thickening
1	ST, A-STT	Persistence	20	31	22	-	19	-	-	Yes
2	ST, A-STT	Persistence	16	29	22	Mild	18	38	Yes [5]	Yes
3	A-STT, LVs	New	21	32	21	Mild	17	37	Yes [6]	Yes
4	ST, A-STT	Persistence	16	28	25	-	16	-	-	-
5	ST, A-STT	New	10	37	20	Mild	19	N/A	Yes [3]	Yes
6	AF, A-STT	Persistence	20	34	29	Severe	20	40	Yes [4]	Yes
7	No	No	15	35	26	Mild	18	N/A	Yes [1]	-
8	ST, A-STT	Persistence	10	32	24	Mild	15	33	Yes [5]	-
9	AV block I	New	10	25	23	Mild	15	N/A	Yes [14]	Yes
10	No	Persistence	18	30	27	Mild	15	N/A	-	-
11	ST, LVs	Persistence	11	32	23	Mild	-	37	Yes [5]	Yes
12	ST, AV block I	New	12	32	19	Mild	16	33	-	-
13	ST, A-STT	Persistence	11	35	24	Mild	18	40	Yes [4]	Yes
14	ST, A-STT	Persistence	10	30	19	Mild	17	28	Yes [1]	Yes
15	ST, A-STT, LVs	Persistence	19	38	28	Mild	17	37	Yes [1]	Yes
16	ST, A-STT, LVs	Persistence	18	30	20	Mild	19	33	Yes [3]	Yes
17	ST, A-STT	Persistence	19	35	25	Severe	16	35	Yes [1]	-
18	ST, A-STT, LVs	Persistence	16	36	26	Mild	18	38	Yes [4]	Yes
19	ST, A-STT, LVs	Persistence	17	26	25	Mild	17	N/A	Yes [5]	Yes
20	ST, A-STT, LVs	Persistence	15	29	23	Mild	17	29	Yes [8]	-
21	A-STT	New	19	38	27	Mild	20	32	-	Yes
22	AF, A-STT, LVs	Persistence	18	30	18	Mild	16	28	Yes [8]	Yes
23	A-STT	Persistence	12	30	22	-	16	-	-	Yes
24	A-STT, LVs	Persistence	16	30	15	Mild	15	40	-	-
25	A-STT	Persistence	14	32	27	Mild	-	35	Yes [3]	Yes
26	ST, A-STT, LVs	New	11	30	23	Mild	17	N/A	Yes [1]	Yes
27	AV block I	New	17	30	23	Severe	17	38	-	-
28	ST, A-STT	Persistence	18	29	25	Mild	16	N/A	Yes [6]	-
29	A-STT	Persistence	14	32	25	Mild	13	32	-	Yes

A-STT, aspecific ST-T abnormalities; AV, atrioventricular; CT, computed tomographic scan of the thorax; IVC, inferior vena cava; LVs, low voltages; RAA, right atrial area; RVEDD, right ventricular end-diastolic diameter; SPAP, systolic pulmonary artery pressure; TAPSE, tricuspid annular plane excursion; TR, tricuspid regurgitation; ST, sinus tachycardia. The number in square brackets refers to pericardial effusion in mm.

**Table 4:** Lung ultrasound findings in patients fully recovered from COVID-19 pneumonia.

	Lung Ultrasound									Fibrosis Bronchiectasis Emphysema (CT)
Pt.	Sliding	B-Lines	Pleural Effusion	Pleural Thickening	Curtain Sign	Right DT Ratio	Left DT Ratio	Right DE (mm)	Left DE (mm)	
1	Yes	-	-	Yes	Yes	1.5	1.7	40	35	-
2	Yes	-	-	Yes	Yes	1.7	2.3	37	39	Yes
3	Yes	-	-	Yes	Yes	2.0	2.0	39	38	Yes
4	Yes	-	-	-	Yes	2.0	1.5	46	40	-
5	Yes	-	Yes	Yes	Yes	1.4	1.1	25	27	-
6	Yes	-	Yes	Yes	-	2.3	2.1	55	47	Yes
7	Yes	-	Yes	Yes	Yes	1.8	2.6	N/A	N/A	Yes
8	Yes	-	-	-	Yes	1.9	1.9	52	40	Yes
9	Yes	-	-	-	Yes	1.6	1.5	35	43	Yes
10	Yes	-	-	-	Yes	1.7	1.7	49	32	Yes
11	Yes	-	Yes	Yes	Yes	2.1	1.6	40	37	-
12	Yes	-	-	-	Yes	1.1	1.1	30	34	Yes
13	Yes	-	Yes	Yes	Yes	1.6	1.5	44	43	Yes
14	Yes	-	-	-	Yes	1.5	1.5	50	50	Yes
15	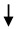 R+L	-	Yes	-	Yes	1.5	1.5	36	41	Yes
16	Yes	-	Yes	Yes	Yes	2.4	2.1	49	32	Yes
17	Yes	-	Yes	Yes	Yes	1.3	1.2	32	42	Yes
18	Yes	-	-	Yes	Yes	1.8	1.6	29	38	Yes
19	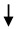 L	-	-	Yes	Yes	1.6	1.6	41	36	Yes
20	Yes	-	-	-	Yes	1.5	1.5	40	41	-
21	Yes	-	-	Yes	Yes	1.7	1.8	40	41	Yes
22	Yes	-	Yes	Yes	Yes	1.6	2.5	51	37	Yes
23	Yes	-	-	Yes	Yes	1.6	1.9	47	41	N/A
24	Yes	-	-	-	Yes	1.6	1.8	38	38	Yes
25	Yes	-	Yes	Yes	Yes	1.7	1.7	47	41	N/A
26	Yes	-	Yes	Yes	Yes	1.7	1.7	40	47	Yes
27	Yes	-	-	-	Yes	2.1	2.1	N/A	N/A	Yes
28	Yes	-	Yes	-	Yes	1.9	2.0	40	30	Yes
29	Yes	-	-	-	Yes	1.9	2.0	37	38	Yes

CT, computed tomographic scan of the thorax; DE, maximum diaphragmatic excursion at total lung capacity; DT, maximum diaphragmatic thickness at total lung capacity; L, left; N/A, not available; R, right; 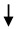, reduction.

## Data Availability

All data are included in this manuscript.
